# Dynamic correlations: exact and approximate methods for mutual information

**DOI:** 10.1093/bioinformatics/btae076

**Published:** 2024-02-10

**Authors:** Kemal Demirtaş, Burak Erman, Türkan Haliloğlu

**Affiliations:** Department of Chemical Engineering, Bogazici University, 34342 Istanbul, Turkey; Polymer Research Center, Bogazici University, 34342 Istanbul, Turkey; Department of Chemical and Biological Engineering, Koc University, 34450 Istanbul, Turkey; Department of Chemical Engineering, Bogazici University, 34342 Istanbul, Turkey; Polymer Research Center, Bogazici University, 34342 Istanbul, Turkey

## Abstract

**Motivation:**

Proteins are dynamic entities that undergo conformational changes critical for their functions. Understanding the communication pathways and information transfer within proteins is crucial for elucidating allosteric interactions in their mechanisms. This study utilizes mutual information (MI) analysis to probe dynamic allostery. Using two cases, Ubiquitin and PLpro, we have evaluated the accuracy and limitations of different approximations including the exact anisotropic and isotropic models, multivariate Gaussian model, isotropic Gaussian model, and the Gaussian Network Model (GNM) in revealing allosteric interactions.

**Results:**

Our findings emphasize the required trajectory length for capturing accurate mutual information profiles. Long molecular dynamics trajectories, 1 ms for Ubiquitin and 100 µs for PLpro are used as benchmarks, assuming they represent the ground truth. Trajectory lengths of approximately 5 µs for Ubiquitin and 1 µs for PLpro marked the onset of convergence, while the multivariate Gaussian model accurately captured mutual information with trajectories of 5 ns for Ubiquitin and 350 ns for PLpro. However, the isotropic Gaussian model is less successful in representing the anisotropic nature of protein dynamics, particularly in the case of PLpro, highlighting its limitations. The GNM, however, provides reasonable approximations of long-range information exchange as a minimalist network model based on a single crystal structure. Overall, the optimum trajectory lengths for effective Gaussian approximations of long-time dynamic behavior depend on the inherent dynamics within the protein's topology. The GNM, by showcasing dynamics across relatively diverse time scales, can be used either as a standalone method or to gauge the adequacy of MD simulation lengths.

**Availability and implementation:**

Mutual information codes are available at https://github.com/kemaldemirtas/prc-MI.git.

## 1 Introduction

Dynamic allostery refers to the transmission of information within a protein from one location to another through correlated thermal fluctuations without noticeable conformational changes, in contrast to the classical concept of allostery which necessitates static conformational changes. The thermal fluctuations of atoms within proteins are typically on the order of Angstroms and can be observed experimentally using techniques such as X-ray scattering and NMR. While X-ray scattering and NMR experiments can accurately determine the mean positions of atoms, as well as the magnitude and anisotropy of fluctuations, measuring the correlations between fluctuations directly has proven elusive. Such correlations have had to be inferred from computational models or limited experimental evidence, primarily using NMR (Stevens *et al.* 2001, Kern and Zuiderweg 2003). Recently, the application of accurate solution X-ray scattering techniques (Meisburger *et al.* 2017, Xu *et al.* 2021) has enabled the determination of correlations between residue fluctuations, which not only confirms the fundamental relationship between correlations and dynamic allostery but also holds promise for future experiments. However, to make further progress in this field, we still require the level of accuracy and detail provided by computational techniques such as molecular dynamics. Molecular dynamics simulations provide a comprehensive description of protein dynamics by generating time series or trajectories of coordinates for each atom. However, equally important to generating these trajectories in functional time frames is extracting useful information from them. Mutual Information is a powerful method that can be used to analyze correlations between protein structure, dynamics, and function. This approach has been successfully applied to explore the communication pathways within proteins and between different protein domains, offering insights into the mechanisms of signal transduction, catalysis, and regulation. For instance, researchers have used mutual information to reveal co-evolving residues in protein families and to identify key residues in protein-protein interactions (Lockless and Ranganathan 1999, Süel *et al.* 2003). The information extracted from mutual information analysis can help advance our understanding of protein dynamics and function, and may aid in the design of novel drugs of allosteric ability and therapeutic interventions (Morcos *et al.* 2011, Shukla *et al.* 2014). In addition, mutual information has been applied to protein design and engineering, guiding the selection of functionally important residues and facilitating the optimization of protein stability and activity (Miyashita and Yonezawa 2021).

While molecular dynamics provides the most thorough depiction of proteins, a critical assessment of the necessary accuracy in relation to simulation length and molecular detail has yet to be conducted. In its full detail, mutual information requires the joint probability of fluctuations of two residues; each residue being characterized in three directions, i.e. a total of six dimensions, necessitating lengthy trajectories in the order of microseconds and beyond. Given this rigorous requirement, different models have been proposed in the literature to simplify the 6D pair joint probability function. These are: (i) The multivariate Gaussian model, where the residue pair's fluctuations are still represented in six dimensions, but the functional form of the function is in terms of the covariance of the fluctuations. The implementation of the Gaussian function—which is a consequence of the central limit theorem—results in a more accurate portrayal of the system, even with shorter trajectories. (ii) The Isotropic Gaussian model, which assumes that fluctuations are isotropic and identical in each coordinate direction. Calculations based on this probability function ignore all cross-correlations. (iii) The Gaussian Network Model. This is a method for determining correlations without the need for molecular dynamics trajectories. Instead, this model uses well-defined crystal structures to build a contact matrix, which can then be inverted to obtain all correlations. Because of the way the model is constructed, the resulting correlations are necessarily isotropic.

Several computational studies have investigated mutual information in proteins using both exact (McClendon *et al.* 2014, Cortina and Kasson 2016, Long and Tian 2016, Singh and Bowman 2017, Sogunmez and Akten 2020, Shao *et al.* 2021, Schneider and Antes 2023), mostly considering the magnitudes of fluctuations, and approximate calculations (Lange and Grubmüller 2006, Jana *et al.* 2011, Rennebaum and Caflisch 2012, Invernizzi *et al.* 2014, Scarabelli and Grant 2014, Köhler *et al.* 2015, Tiberti *et al.* 2015, Ahmed and Barakat 2017, Milenkovic and Bondar 2018, Zhang *et al.* 2018, Foster *et al.* 2021, Payne *et al.* 2022, Tekpinar and Yildirim 2022). By relying on the three approximations, calculations requiring shorter simulations or only static crystal structures may become possible. However, since correlations play a crucial role in determining protein function and given the possibility of new experimental studies on this topic, it is crucial to obtain an accurate estimate of the validity limits of these approximations.

**Figure 1.**
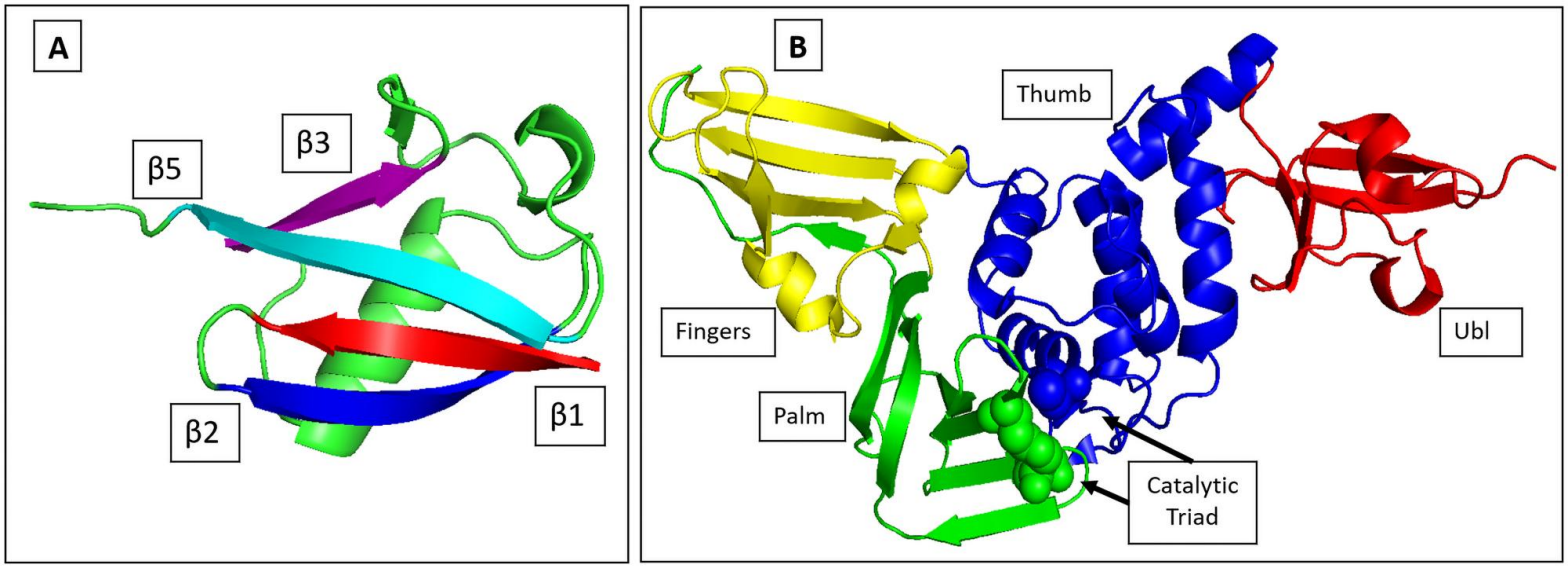
(A) Ubiquitin (PDB: 1UBQ) with β1-β4 colored. (B) PLpro (PDB: 6WX4) with its subdomains colored and catalytic triad shown in spheres

This paper has two main objectives. Firstly, we aim to establish a tentative lower limit for the required length of MD trajectories in order to achieve a desired level of accuracy or at least posing this as an important criterion by using one millisecond and hundred microseconds molecular dynamics trajectories of two extensively studied proteins Ubiquitin and PLpro as reference points (Fig. 1). The choice of these two proteins for mutual information analysis stem from structural characteristics that could exhibit notable distinct correlation patterns and also accessibility of long MD simulation trajectories and availability of functional information, in addition to their biological significance. The molecular dynamics trajectories that we used for our analysis were kindly given to us by D.E. Shaw Research group. Secondly, we aim to compare the accuracy levels of different approximations as a function of trajectory length. Here, while noting that different time windows possibly reveal various physically plausible correlations, we assume the longest MD trajectories represent the ground truth for long range dynamic correlations. Our overall goal is to show that dynamic allostery is a phenomenon that can be quantified computationally, and that its characterization can be done quickly and accurately.

## 2 Materials and methods

### 2.1 Molecular dynamic data

1 ms ubiquitin (Lindorff-Larsen *et al.* 2016) (PDB code: 1UBQ) and 100 µs PLpro SARS-CoV-2 (Shaw 2020) (PDB code: 6wx4 with the ligand removed) molecular dynamic simulation trajectories are utilized for mutual information analysis of exact anisotropic and multivariate and isotropic Gaussian calculations. These crystal structures are used for the GNM based mutual information calculations.

The fluctuation vector for two residues is defined with a 6D vector as

(1) $$\Delta R\left(t\right)=\mathrm{col}\left[{\Delta R}_{i}\left(t\right)\;{\Delta R}_{j}\left(t\right)\right]=\mathrm{col}\left[{\Delta X}_{i}\left(t\right)\;{\Delta Y}_{i}\left(t\right)\;{\Delta Z}_{i}\left(t\right)\;{\Delta X}_{j}\left(t\right)\;{\Delta Y}_{j}\left(t\right)\;{\Delta Z}_{j}\left(t\right)\right]$$

where, col denotes the column.

### 2.2 Shannon entropy

Because of thermal fluctuations, every time series possesses a degree of uncertainty, which can be quantified by the Shannon entropy $-〈\log\left[p\left({\Delta R}_{i}\left(t\right)\right)\right]〉$ where $p\left({\Delta R}_{i}\left(t\right)\right)$ is the probability of fluctuation of atom i at time t, and the angular brackets denote average over the full trajectory. Shannon initially employed a logarithm to base 2 to quantify entropy. However, Callen *et al.* (1985) subsequently demonstrated that multiplying by the Boltzmann constant and utilizing natural logarithms aligned with the thermodynamic entropy of a system. Numerous information theoretic measures can be defined both within the trajectory of a single residue and between the trajectories of different residues, all derived from Shannon entropy, such as mutual information, conditional entropy, entropy transfer, and interaction information. The uncertainty in either in the incomplete sampling or the limitation of the physical model is the fundamental element that needs to be considered while developing new functions. These measures provide countless opportunities to enhance our comprehension of allostery. For instance, mutual information is a critical concept in allostery, as it measures the reduction in uncertainty, or entropy, in the trajectories of a pair of residues caused by their correlated movements.

#### 2.2.1 Exact anisotropic mutual information

The exact definition of mutual information $I\left({\Delta R}_{i},{\Delta R}_{j}\right)$ is given by the expression

(2) $$I\left({\Delta R}_{i},{\Delta R}_{j}\right)=〈\log\frac{p\left({\Delta R}_{i},{\Delta R}_{j}\right)}{p({\Delta R}_{i}){p(\Delta R}_{j})}〉$$

where, the time arguments of ΔR are not shown for simplicity. Here, $p({\Delta R}_{i},{\Delta R}_{j})$ is the joint probability of having fluctuations ${\Delta R}_{i}$ and ${\Delta R}_{j}$of residues *i* and *j*, respectively and p(ΔR)’s are the marginal probabilities. In terms of Cartesian coordinates, each fluctuation ΔRhas three components and the probability in the numerator of the logarithmic term in Equation (2) is a 6D function. $p\left(\Delta X_{i},\Delta Y_{i},\Delta Z_{i},\Delta X_{j},\Delta Y_{j},\Delta Z_{j}\right).$ This represents the most detailed form for the joint probability of fluctuations of two residues, and accounts for contributions arising from anisotropies of fluctuations addressed by unequal fluctuations along different coordinate directions. As discussed in the Introduction section, an accurate characterization of the 6D function requires a large number of data points. Therefore, certain simplifications are applied to the general expression. Below, we discuss four approximations to Equation (2). The exact form of mutual information was evaluated by Lange and Grubmuller and calculations were based on two trajectories 200 ns and 117 ns long. While they were predominantly interested in the relationship of his form to Pearson’s coefficient and to a generalized representation, convergence of the exact mutual information with trajectory length, which is the main aim of the present paper, was not investigated.

#### 2.2.2 The multivariate Gaussian approximation

This approximation is based on the joint probability

(3) pijΔR=2π-n2detΔRΔRT-12exp-12ΔRTΔRΔRT-1ΔR

where det represents the determinant, superscript T represents the transpose, angular brackets denote the time average. We show in the Supplementary Material section that using the relationship of mutual information to entropy, the expression of mutual information for multivariate Gaussian approximation is

(4) $$I\left({\Delta R}_{i},{\Delta R}_{j}\right)=-\frac{1}{2}l\mathrm{og}\left(\frac{\det〈\Delta R{\Delta R}^{T}〉}{\det〈{\Delta R}_{i}{\Delta R}_{i}^{T}〉d\mathrm{et}〈{\Delta R}_{j}{\Delta R}_{j}^{T}〉}\right)$$

The multivariate approximation reduces the problem to the calculation of the 6x6 correlation matrix, $〈\Delta R\Delta R^{T}〉$, which can easily be evaluated from molecular dynamics trajectories. The Gaussian approximation to the exact representation of mutual information was first introduced and named Linearized Mutual Information by Lange and Grubmuller.

#### 2.2.3 Exact isotropic mutual information

The exact isotropic model relies on the probability distribution of magnitudes of ΔR vectors to be obtained from molecular dynamics trajectories. The joint probability of fluctuations of two residues is formed as $p\left({|\Delta R}_{i}|\;|{\Delta R}_{j}|\right)$, which excludes potential anisotropic contributions. Equation (2) is rewritten as

(5) $$I\left({\Delta R}_{i},{\Delta R}_{j}\right)=\;〈\frac{p\left({|\Delta R}_{i}|,|{\Delta R}_{j}|\right)}{p\left(\left|{\Delta R}_{i}\right|\right)\times p\left(\left|{\Delta R}_{j}\right|\right)}〉$$

#### 2.2.4 The isotropic Gaussian approximation (zero off-diagonal approximation)

The off-diagonal elements of the correlation matrix $〈\Delta R\Delta R^{T}〉$ provide information about the anisotropic motions in the system. For example, $〈\Delta X_{i}\Delta Y_{j}〉$ gives the correlation between the motions of the ith residue along the X-direction and of the jth residue along a perpendicular direction. When the off-diagonal terms are ignored, the matrix $〈\Delta R\Delta R^{T}〉$ may be replaced by$〈\Delta R^{T}\Delta R〉$. Equation (4) then takes the form

(6) $$I\left({\Delta R}_{i},{\Delta R}_{j}\right)=-\frac{1}{2}l\mathrm{og}\left(1-\frac{{〈{\Delta R}_{i}^{T}{\Delta R}_{j}〉}^{2}}{〈{{(\Delta R}_{i})}^{2}〉〈{({\Delta R}_{j})\;}^{2}〉\;}\right)$$

The difference between results of isotropic and anisotropic methods will be at the regions of the protein which are more affected by correlation of off-diagonal terms. If there is significant correlation between off-diagonal terms of residue pair *i* and *j*, multivariate Gaussian will better represent their concerted motion. If the correlation in their off-diagonal term is low, it does not necessarily mean that anisotropy is absent. However, the Gaussian isotropic method, using the zero off-diagonal approximation, can still adequately explain the concerted motions of i and j.

#### 2.2.5 The Gaussian network model, GNM

GNM is a coarse-grained method where each residue is reduced to its alpha carbon and each alpha carbon is connected to its neighboring alpha carbons by linear springs. Represented as an elastic mass-and-spring network, Kirchhoff’s (connectivity) matrix, $\Gamma_{ij}$, encodes topological information of the protein and it is formed as

(7) Γij=-1 if i ≠j and Rij≤rc0 if i ≠j and Rij>rc-∑k=1, k≠iNΓik if i=j 

where $R_{ij}$ and $r_{c}$ represent distance between residues *i* and *j* and a certain pre-determined cutoff radius, *N* is the number of residues. Mutual information identical to Equation (6) could thus be derived using GNM (Haliloğlu *et al*. 2022) where covariance matrix could be calculated as follows

(8) $$〈\Delta R_{i}\;\Delta {R_{j}}^{T}〉=\;\frac{3k_{b}T}{\gamma }{\left({\Gamma_{\mathrm{ij}}}^{-1}\right)}_{\mathrm{ij}}=\;\frac{3k_{b}T}{\gamma }{\left[U\Lambda^{-1}U^{T}\right]}_{\mathrm{ij}}$$

where *k_b_* is Boltzmann constant, *T* is absolute temperature, γ is force constant. **U** and **Λ** are obtained by eigenvalue decomposition of Kirchhoff connectivity matrix **Γ**. **U** is unitary matrix of eigenvectors and **Λ** is diagonal matrix of eigenvalues.

The GNM represents protein motions as a sum of N-1 dynamic modes. Each mode captures a specific range of timescales: the first includes the slowest (most dominant) motions, while subsequent modes progressively exclude these slowest motions and focus on faster dynamics. In other words, considering only subsets of 2 or more modes ignores the slowest, large-scale motions and reveals details of faster subtimescales within the overall dynamics.

#### 2.2.6 Comparison of MI profiles and grid size

To compare the MI values across increasing trajectory lengths using various approximations and the exact anisotropic MI values from the longest trajectory, we used two criteria: root mean square deviation (RMSD) and correlation coefficient. We conducted these calculations in detail for two representative functional residues, residue 53 of Ubiquitin and residue 111 of PLpro. These residues are selected for their functional importance; residue 53 is one of the residues of the allosteric switch residues with residues 24/52-governing the opening of the protease binding region in Ubiquitin and Residue 111 is as a member of the catalytic triad of PLpro is directly involved in catalytic activities. Specifically, we compared the MI profiles of these residues. These MI profiles illustrate the extent of correlation and information sharing between these residues and others. We repeated the calculations also for all residues in the respective structures.

In this context, we calculated the RMSD between the exact anisotropic, exact isotropic, multivariate Gaussian, and isotropic Gaussian MI values of shorter trajectories and the exact anisotropic MI values of the longest trajectory, spanning Sections 3.1–3.4. Lastly, in Section 3.5, we determine the RMSD between the MI values derived from GNM 1-all, including all modes of motion, and the exact anisotropic MI values of trajectories at different lengths.

Correlation coefficients are a measure for assessing the similarity in shape between two MI profiles. Even if MI values have not yet reached convergence, essential features of the MI profile can emerge earlier. To compute these coefficients, we used *corr* function of MatlLab, which computes the Pearson correlation coefficient between the two MI profiles. Correlation coefficient is calculated between MI profiles of different trajectories of the same residue. Sections 3.1–3.4 exhibit the correlations coefficients for the identical cases described in RMSDs.

Protein behavior particularly in short trajectories can vary due to limited statistical sampling. To address this bias, we selected multiple segments from different points within the longest trajectory (Supplementary Tables S1 and S2). We calculated the mutual information of each residue pair for each segment independently and then average these values to determine the mutual information for that specific trajectory length (Supplementary Tables S3 and S4). These average MI values are used for the calculations of the RMSDs and correlation coefficients.

Grid size refers to the number of intervals each time series is divided for calculation of exact form of mutual information. In the exact anisotropic method, distinct contributions of each dimension of fluctuations are considered and ΔX, ΔY, and ΔZ are histogrammed separately whereas only delR is histogrammed in the exact isotropic method. In order to determine grid size, firstly, each trajectory length is evaluated by between multivariate Gaussian and exact anisotropic MIs at different grid sizes. Then, the grid size that minimizes the RMSD is found suitable for the description of information sharing of that trajectory.

It is crucial to recognize that proteins exhibit a spectrum of functional motions essential for their biological activities, ranging from rapid local fluctuations (picoseconds to nanoseconds) to slower global conformational changes (microseconds to milliseconds). These motions exist as a nexus of overlaid dynamics across diverse time scales. Therefore, the selection of grid sizes in exact mutual information (MI) methods is paramount, as it directly impacts the model's ability to represent this heterogeneity of protein movements. Choosing overly small grids risks failing to detect functionally important correlations due to insufficient detail. Conversely, using larger grids than necessary leads to overfitting, again precluding the capture of functionally relevant relationships. Nonetheless, the optimal grid size depends on the characteristic functional motions within the timescale covered by the trajectory. This implies that the perception of faster versus slower movements and their superimposition is relative to each time window, and optimal grid sizes across different windows may not correspond to functionally similar timescales.

## 3 Results

Below we present the results of MI analysis using 1 ms and 100 microseconds MD simulation trajectories, respectively, of Ubiquitin and PLpro, and discuss their convergence features. In the main body of the paper the MI behavior of selected functional residues 53 of Ubiquitin and 111 of PLpro are presented in detail. We also provide and discuss the results for all residues in 2D MI maps.

### 3.1 Dependence of exact anisotropic MI values on trajectory length

**Figure 2.**
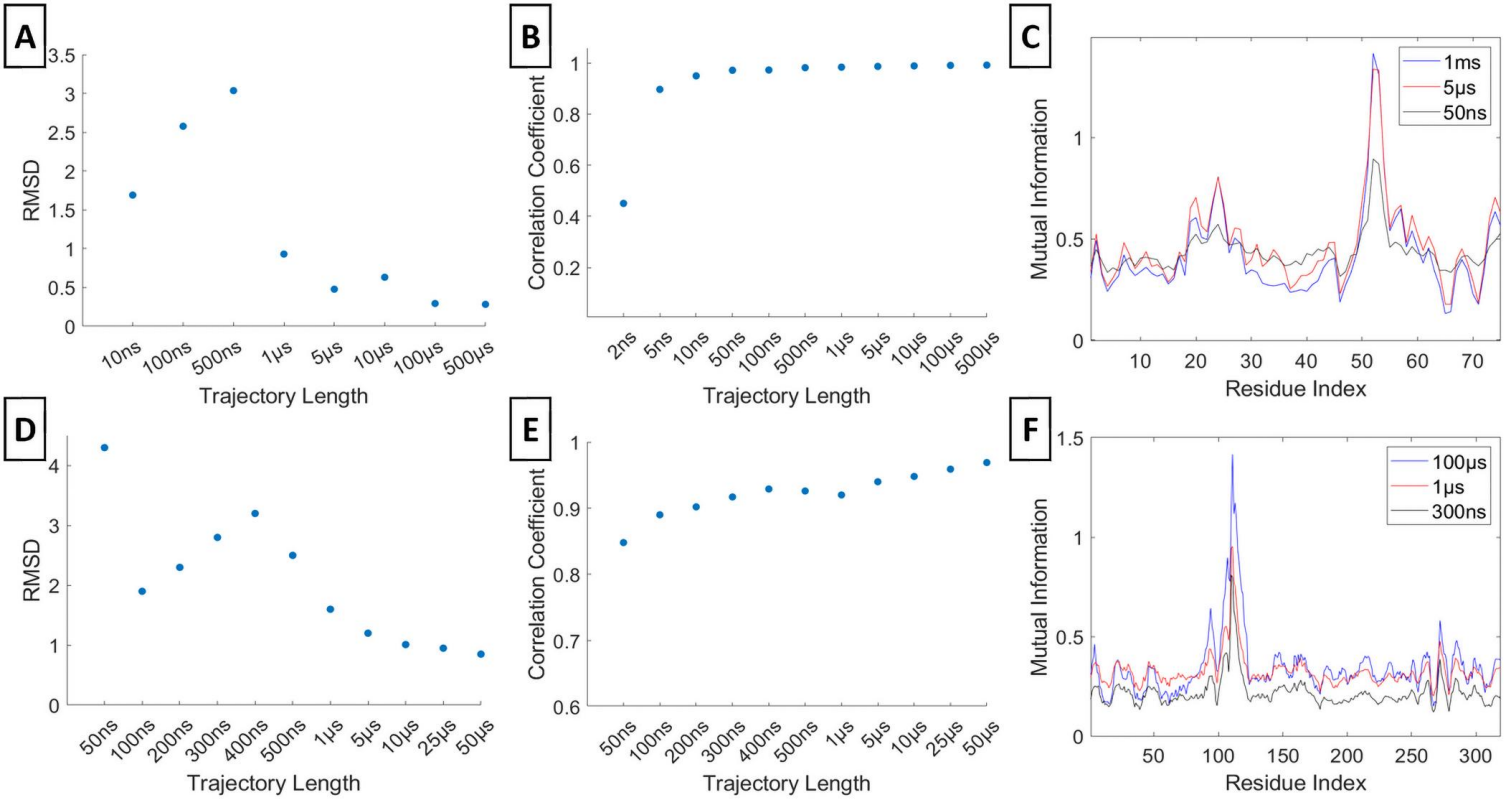
Exact Anisotropic MI. RMSD and correlation coefficient values between the exact anisotropic MI profile at various trajectory lengths and the maximum trajectory length are respectively shown for residue 53 of Ubiquitin (A, B) and residue 111 of PLpro (D, E). The exact anisotropic MI profiles at selected convergent time points and at the maximum trajectory lengths are respectively in (C) and (F). The maximum trajectory length is 1 m for Ubiquitin and 100 µs for PLpro

We assessed the exact MIs [Equation (2)] for varying trajectory lengths and observed their convergence toward the longest trajectories. Specifically, we determined the minimum trajectory lengths necessary to accurately capture the MI profiles observed in the longest trajectory for residue 53 of Ubiquitin and residue 111 of PLpro (Fig. 2). We computed the RMSD between the MI profiles from each trajectory length and the longest trajectory (Panels A and D). The RMSDs converges at approximately 5 µs for Ubiquitin and 1 µs for PLpro, indicating these as the minimum trajectory lengths needed to represent the MI profile of the longest trajectory. The correlation coefficients (Panels B and E) demonstrates that around 50 ns for Ubiquitin and 300 ns for PLpro suffice to exhibit exact MI profiles with similar characteristics to those of the longest trajectory (C and F).

### 3.2 Accuracy of multivariate Gaussian model in representing MI profiles

**Figure 3.**
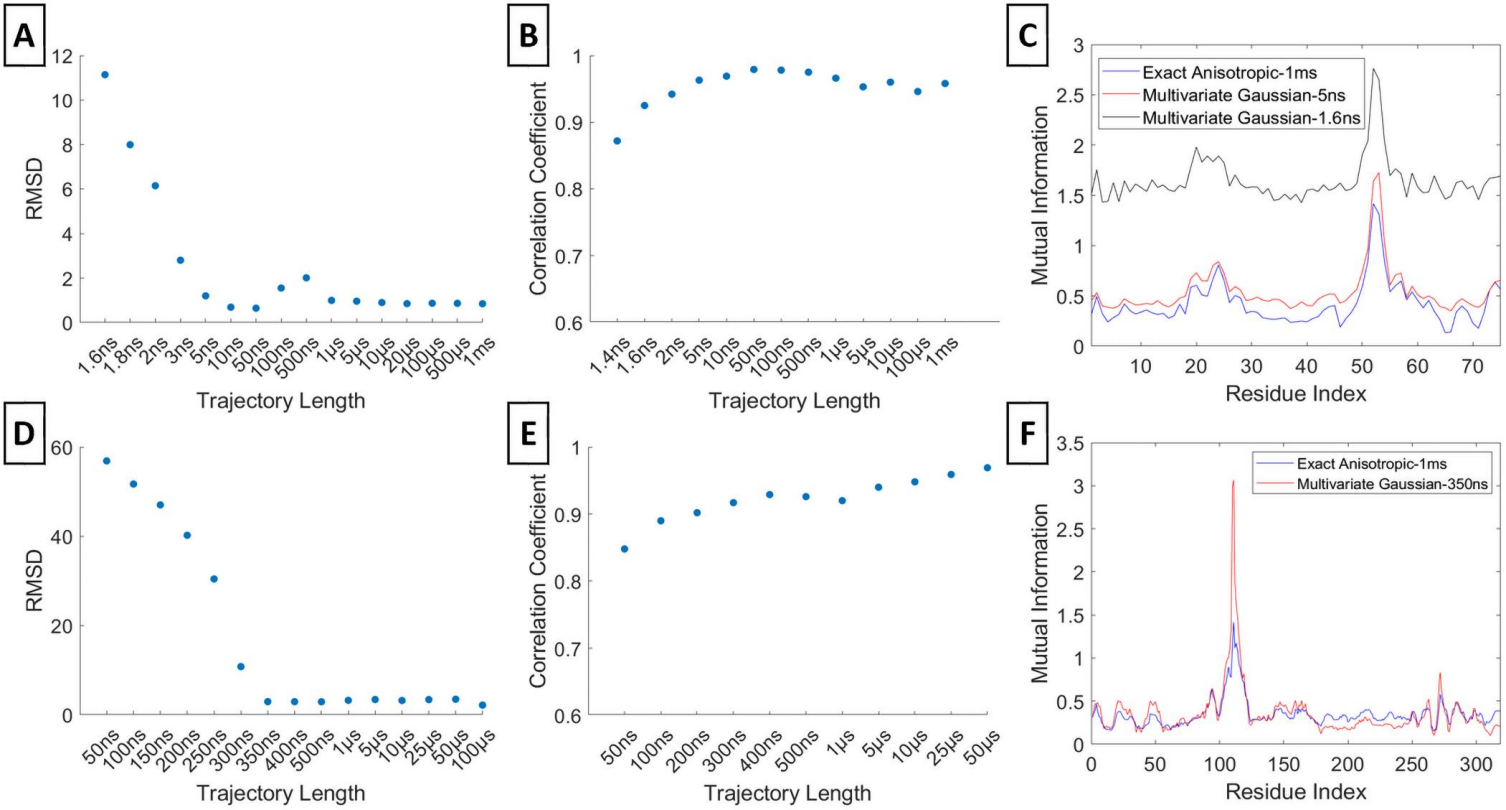
Multivariate Gaussian MI. RMSD and correlation coefficient values between the multivariate Gaussian MI profiles at various trajectory lengths and the exact anisotropic MI profile at the maximum trajectory length are respectively shown for residue 53 of Ubiquitin (A, B) and residue 111 of PLpro (D, E). The multivariate Gaussian MI profiles at selected convergent time points and the exact anisotropic MI at the maximum trajectory lengths are respectively in (C) and (F)

Subsequently, we explored the application of the multivariate Gaussian model in representing the MI profiles accurately for the same Ubiquitin and PLpro residues (Fig. 3). We generated multivariate Gaussian MI profiles for varying trajectory lengths and compared them with the exact anisotropic MIs of the longest trajectory (Panels A and D). Based on the RMSDs, the minimum trajectory length required for accurate representation by the multivariate Gaussian model is 5 ns for Ubiquitin and 350 ns for PLpro (Panels A and D). In addition, the correlation coefficients converge at 350 ns for PLpro, whereas 1.6 ns is sufficient for Ubiquitin to exhibit certain characteristics of the longest trajectory (Panels B and E). Panels C and F depict the minimum necessary multivariate Gaussian profiles alongside the longest exact MI profile. Also, it shows the discrepancy between MI values before and after convergence. Overall, the results indicate that the multivariate Gaussian model can effectively represent the MI profiles with considerably shorter trajectory lengths for Ubiquitin and PLpro.

### 3.3 Dependence of exact isotropic MI values on trajectory length

**Figure 4.**
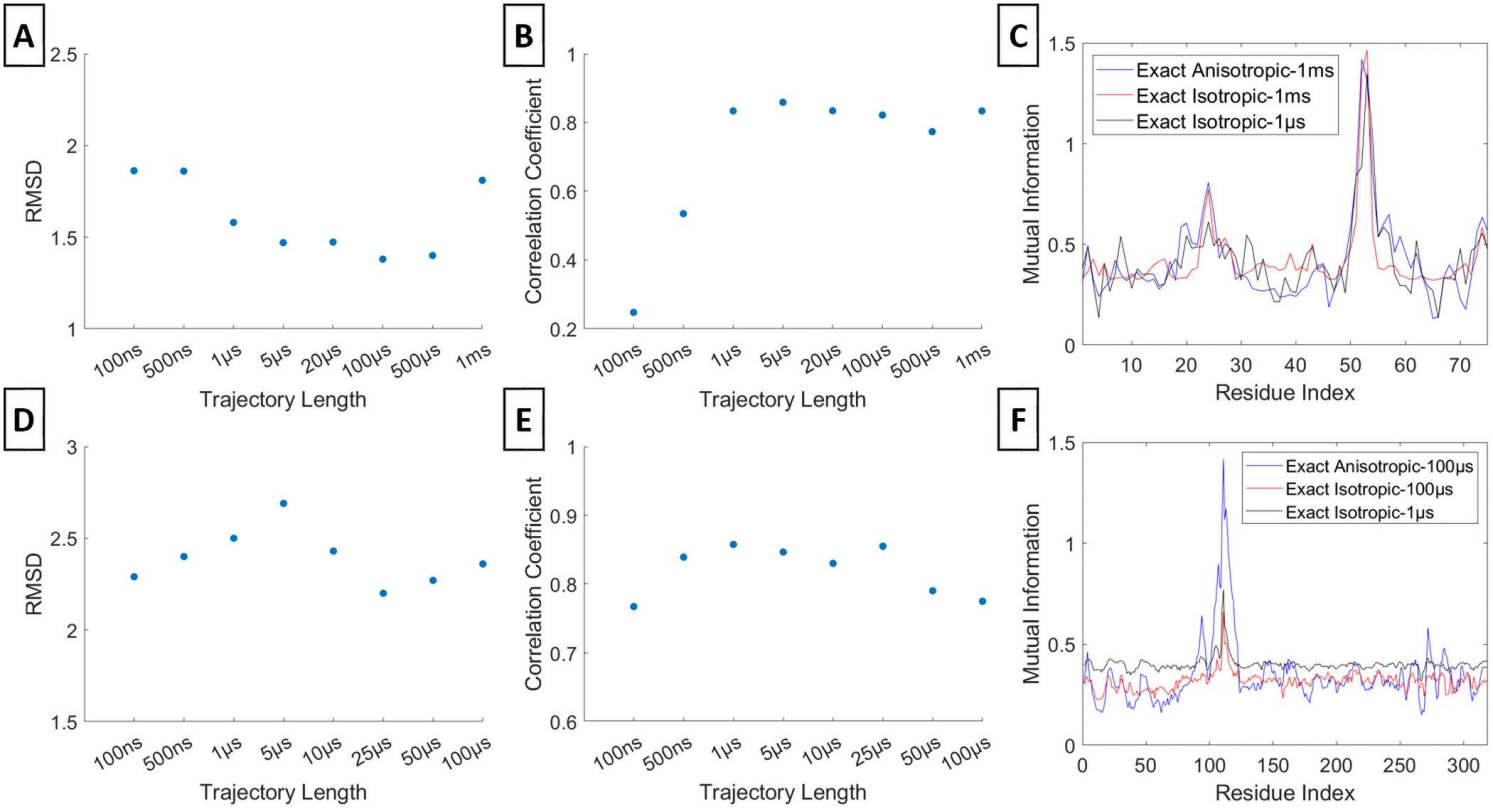
Exact Isotropic Gaussian MI. RMSD and correlation coefficient values between the exact isotropic MI profiles at various trajectory lengths and the exact anisotropic MI value at the maximum trajectory length are respectively shown for residue 53 of Ubiquitin (A, B) and residue 111 of PLpro (D, E). The exact isotropic MI profile at a selected convergent time point and the isotropic and exact anisotropic MI profiles at the maximum trajectory lengths are respectively in (C) and (F)

In this context, we conducted a comparison between the exact isotropic and exact anisotropic MI profiles (Fig. 4). For both Ubiquitin's residue 53 and PLpro's residue 111, the RMSDs display fluctuations rather than convergence, in contrast to the behavior observed in the exact anisotropic and multivariate Gaussian MI profiles (Panels A and D). Similarly, the correlation coefficients for PLpro exhibit fluctuations, while those for Ubiquitin converge around the 1 µs (Panels E and B, respectively). Although the exact isotropic MI profiles manage to capture significant peaks, they miss certain local peaks (Panels C and F).

### 3.4 Accuracy of isotropic Gaussian model in representing MI profiles

**Figure 5.**
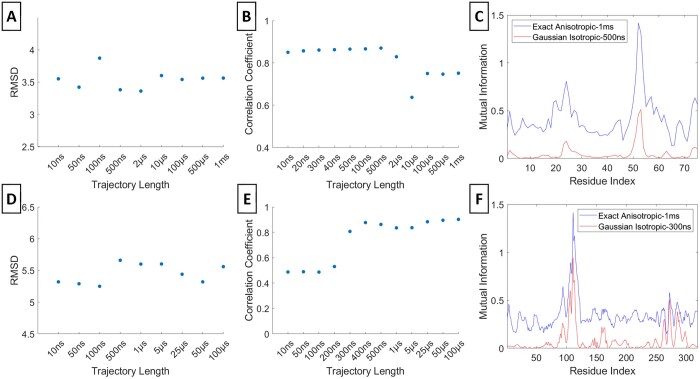
Isotropic Gaussian MI. RMSD and correlation coefficient values between the isotropic Gaussian Gaussian MI profiles at various trajectory lengths and the exact anisotropic MI profile at the maximum trajectory length are respectively shown for residue 53 of Ubiquitin (A, B) and residue 111 of PLpro (D, E). The isotropic Gaussian MI profile at selected convergent time point and the exact anisotropic MI at the maximum trajectory lengths are respectively in (C) and (F). The maximum trajectory length is 1 m for Ubiquitin and 100 µs for PLpro

In addition, we also had an assessment of the isotropic Gaussian assumption's effectiveness in approximating the precise anisotropic MI profiles for the same residues in Ubiquitin and PLpro (Fig. 5). The RMSDs between the Gaussian isotropic and the anisotropic exact MI profiles from the longest trajectory do not exhibit convergence (Panels A and D). Although the correlation coefficients exhibit fluctuations for Ubiquitin, they do eventually converge around 300 ns for PLpro (B and E). In contrast to the multivariate Gaussian approach, the isotropic Gaussian MI profiles, determined by RMSDs and the correlation coefficients, highlights that the isotropic model falls short in representing the exact anisotropic MI profile (Panels C and F). This limitation primarily stems from the isotropic Gaussian model's omission of orthogonal correlations. However, the isotropic Gaussian supersedes the exact isotopic model by revealing some profile characteristics similar to the exact anisotropic and multivariate Gaussian models.

### 3.5 Accuracy of GNM in representing MI profiles

**Figure 6.**
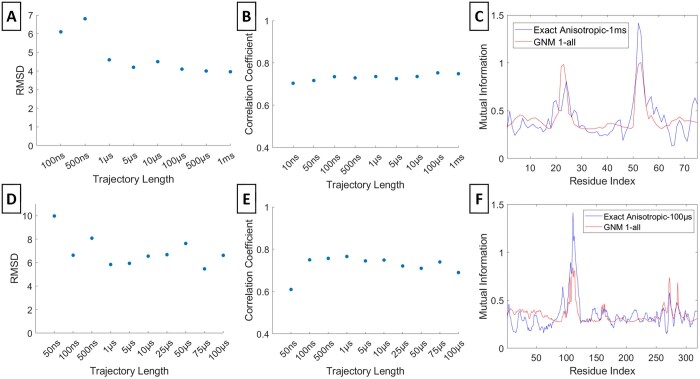
GNM MI. RMSD and correlation coefficient values between the GNM MI profiles and the exact anisotropic MI profile at various trajectory lengths are respectively shown for residue 53 of Ubiquitin (A, B) and residue 111 of PLpro (D, E). The Gaussian MI profile and the exact anisotropic MI at the maximum trajectory length are respectively in (C) and (F). The maximum trajectory length is 1 m for Ubiquitin and 100 µs for PLpro

We have also calculated the MI profiles using GNM on the apo X-ray crystal structures (PDB code: 1ubq for Ubiquitin & PDB code: 6wx4 with the ligand removed for PLpro) and compared them with the exact anisotropic MI profiles (Fig. 6). The RMSDs observed between the GNM MI profiles and the exact anisotropic MI profiles at various trajectory lengths exhibit a decrease as the trajectory length extends (Panels A and D). Correspondingly, the correlation coefficients follow an increasing pattern, highlighting the GNM's capability in accurately representing the long-term behavior (Panels B and E). When comparing the GNM profiles to the longest exact MI profiles, it is evident that the GNM effectively captures the major peaks (Panels C and F). These findings suggest that the GNM holds promise as a valuable tool for representing MI profiles using a single conformation.

### 3.6 Trajectory length and grid size in mutual information analysis

The MI profiles are notably influenced by the trajectory length, as each length corresponds to a specific time window that reveals distinct correlations. In our pursuit to uncover long-range communication patterns, the longest available trajectory serves as a reference point, a ground truth to be achieved. Consequently, both the minimum trajectory length and grid size emerge as pivotal parameters governing the exploration of MI profiles. The MI results for short trajectories are obtained by averaging multiple MI results from trajectories of the same length, selected from various sections of the longest trajectory. Our assumption is that the longest trajectory represents the ground truth, even though simulations in the past were notably much shorter. Tiberti *et al.* studied mutual information in the context trajectory lengths, highlighting sufficiency of shorter simulations in multiple replicas to approximate long time behavior. The maximum length considered was one microsecond. On the other hand, it was previously suggested the elastic network model based MI can provide an accurate estimation of dynamic correlations and linear MI by using several microsecond simulations (Tekpinar and Yildirim 2018).

It's crucial to recognize that while these insights pertain to the systems we've examined, they underscore the necessity of tailoring grid size for exact calculations and trajectory length for both exact and approximate calculations on a per-case basis. This approach ensures that the analysis effectively captures information exchange and furnishes pertinent insights into the system at hand. For instance, factors such as protein size, structural and dynamic complexity, and anisotropy merit consideration when determining the appropriate trajectory length. To illustrate, here a smaller protein like Ubiquitin warrants a shorter minimum trajectory to capture long-time dynamic behavior. Conversely, a larger, more intricate multidomain protein like PLpro could necessitate a longer minimum trajectory length for accurate representation of long-time behavior. Furthermore, the presence of anisotropy in the protein's shape, as more in PLpro, may further underscore the complexity of the interplay between these parameters.

### 3.7 Case-specific insights for allosteric behavior in Ubiquitin and PLpro

Figures 7 and 8, respectively, show MI values of residue 53 of Ubiquitin and residue 111 of PLpro with other residues color coded on the structure in the exact and approximated calculations. Residue 53 of Ubiquitin and residue 111 of PLpro were selected for the analysis in detail so far but information sharing of all residues also included below Supplementary Figs S1–S7, based on the time points of convergence in Figs 2, 3, and 6, as well verified in Supplementary Figs S5–S7.

**Figure 7.**
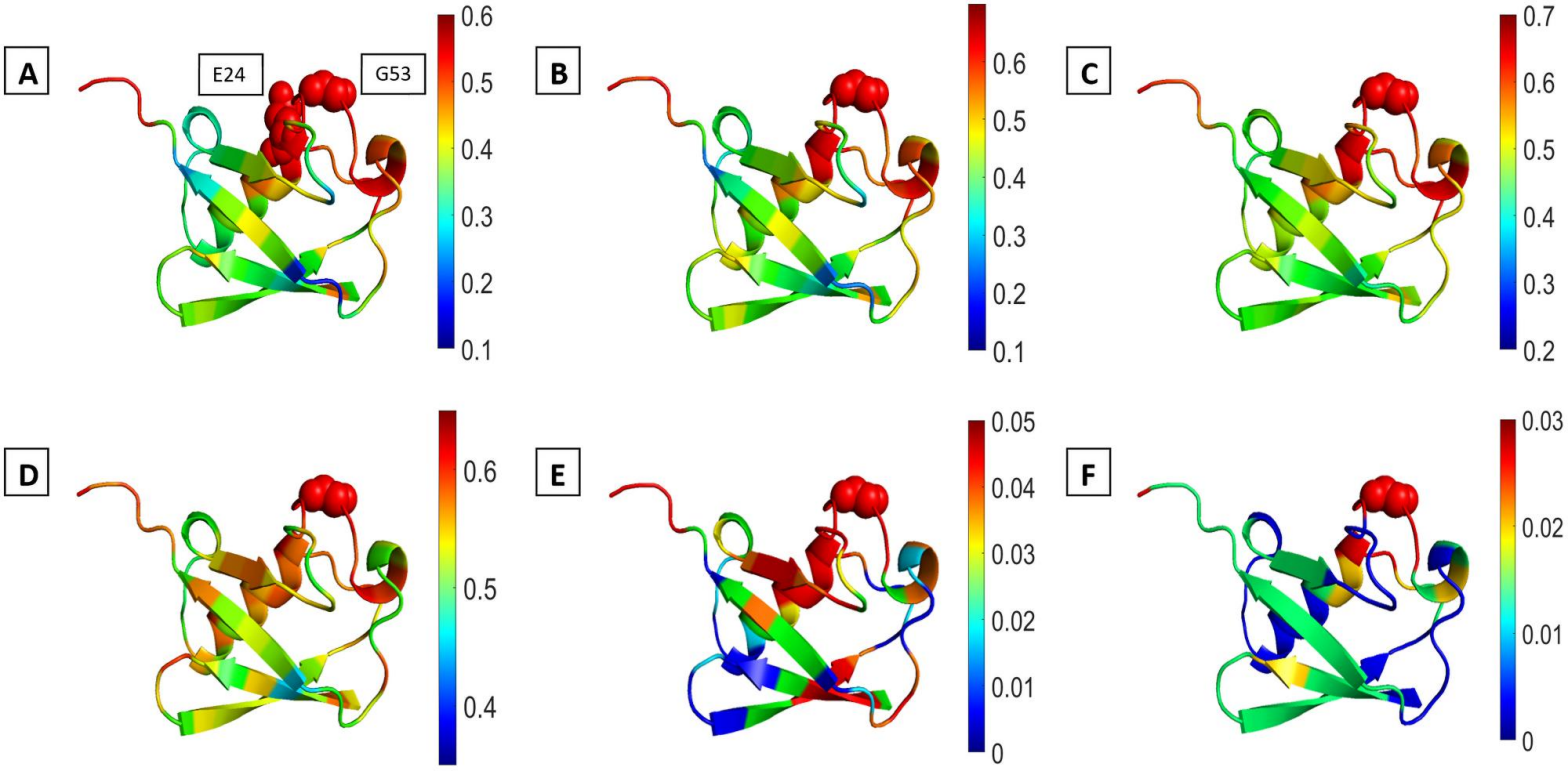
MI profiles of residues 53 of Ubiquitin color-coded on the structure (1UBQ). The exact anisotropic MI of 1 m (A), the exact anisotropic MI of 5 µs trajectory (B), the multivariate Gaussian MI evaluation of 5 ns trajectory (C), the exact isotropic MI of 1 µs trajectory, (E) the isotropic Gaussian MI of 500 ns (E), GNM 1-all MI (F)

**Figure 8.**
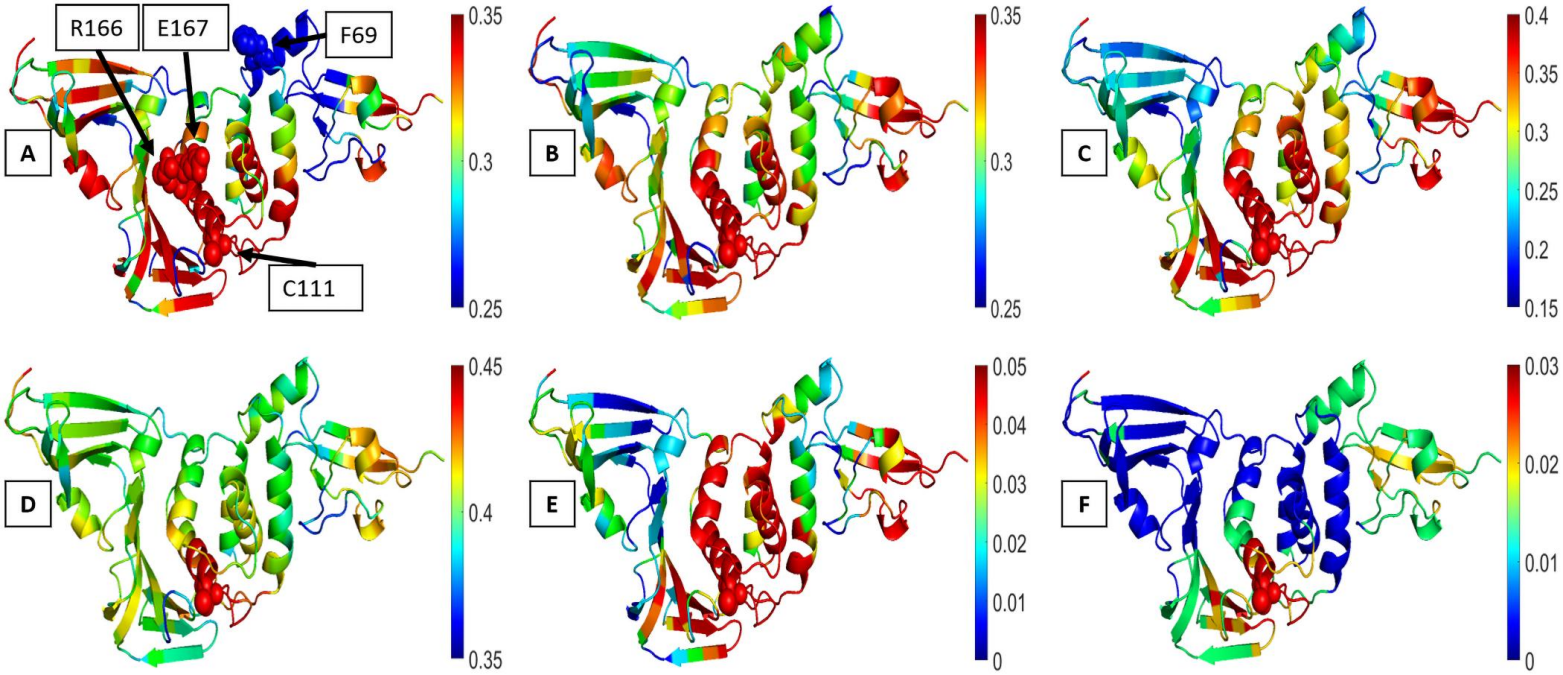
MI of PLpro color-coded on the structure (6WX4). The anisotropic MI of 100 µs (A), the exact anisotropic MI of 1 µs (B), the multivariate Gaussian MI of 350 ns (C), the exact isotropic MI of 1 µs trajectory (D), the isotropic Gaussian MI of 300 ns (E), GNM (1-all modes) MI (F)

#### 3.7.1 Ubiquitin

The D52-G53 peptide bond within Ubiquitin functions as a pivotal allosteric switch, prominently participating in concerted motion dynamics. Its interaction with E24 intricately oversees the precise regulation of the opening mechanism within the USP binding interface. The conformational state of this peptide bond, whether in-state or out-state, governs the overall contraction or expansion of the ubiquitin structure. This regulatory process hinges on the interplay between this allosteric switch and the β1-β3 and β5 strands encircling the USP binding region (Smith *et al.* 2016). Significant interactions between E24 and the D52/G53 region are strongly reflected in the MI values (Fig. 7). Notably, the exact anisotropic MI profiles of 1 ms for both E24 and G53 exhibit distinct peaks at residues Q2, T7, F45, K63, and L69. These peaks suggest potential involvement in the binding of diverse USPs. These identifiable peaks are consistently evident in shorter exact anisotropic and multivariate MI profiles. These residues are also somewhat identifiable in the isotropic Gaussian and GNM mutual information (MI) profiles, although less prominently.

On the other hand, the 2D Mutual Information (MI) maps present a comprehensive overview of the highly information-sharing residues within the whole Ubiquitin structure (Supplementary Fig. S1). In a study referenced (Fenwick *et al.* 2011), a correlation network in Ubiquitin was identified to span a distance of 15 Å, encompassing residues 5–6 (β1 strand), 13–14 (β2 strand), 44–45 (β3 strand), and 67–68 (β5 strand) positioned on the β1-β3 and β5 strands. Both the exact and multivariate Gaussian MI maps depict information sharing patterns among residues: 5–6 and 44, 5–6 and 68, 13–14 and 44, 13–14 and 68, and 44–45 and 68. Notably, the exact anisotropic and multivariate MI maps reveal distinct individual correlations within these residues. However, the Gaussian Network Model (GNM) MI map indicates a regional correlation between the β2 and β5 strands, while not displaying a similar level of correlation between β1 or β2 with β3. Nevertheless, these correlations are partially evident in GNM 3-all modes, 4-all modes, and 5-all mode MI maps (Supplementary Fig. S2). On another note, the isotropic Gaussian MI map inadequately captures these specific correlations, whereas the exact isotropic map closely resembles the exact anisotropic map. Considering that both E24 and G53 share information with certain residues in this network or their neighboring residues, the role of the D52-G53 peptide bond as an allosteric switch is substantiated. This switch regulates the expansion/contraction of the protein structure through coordinated motions within this network.

Overall, the exact anisotropic and multivariate Gaussian MI maps are almost the same but exact isotropic and isotropic Gaussian MI map fails to capture some of the correlations. 1-all modes map of GNM shows collective correlations of some secondary structure elements, while the subsets of modes of GNM render others. Since GNM is better at revealing long-time behavior albeit being isotropic, and these collective correlations of secondary structural elements are not prominent in MI maps of 1 ms trajectory, it may be argued that some correlations in Ubiquitin covers a time frame that is longer than 1 ms. Despite of these differences, some regions appear at both exact anisotropic and GNM 1-all maps. For example, correlations of β1- β2 strands with α1 helix appears at both exact and GNM 1-all maps. Correlation of G53 with residues 5–12 is remarkable in GNM 1-all and also appears in the exact anisotropic maps even though it is less highlighted. On the other hand, the correlation of the tail part with some regions appears at both exact anisotropic and GNM 2-all and 3-all MI maps. Secondly, since Ubiquitin is in a globular shape, inconsistencies that can result from isotropic assumption of GNM are possibly not in question. Such similarities are less observed in PLpro (below). This is also expected because the simulation time of Ubiquitin is much longer, which means that the likelihood of capturing slowest motion is comparatively higher.

#### 3.7.2 PLpro

PLpro features four subdomains: ubiquitin-like (Ubl) (residues 1–60), thumb (residues 61–178), palm (residues 179–239), and fingers (residues 240–315). The catalytic triad, a cysteine-based arrangement (C111, H272, and D286), lies at the thumb-palm subdomain junction, serving as a common hydrolase enzyme motif. Viral polyproteins interact with C111, leading to cleavage and the subsequent generation of non-structural proteins crucial for viral replication (Henderson *et al.* 2020). Moreover, PLpro engages with host Ubiquitin and ISG15 to dampen the immune response against the virus. These interactions occur at S1–S4 binding sites. Among these, S2 is situated at the thumb subdomain, while the rest are spread across the thumb, fingers, and palm subdomains. Key residues, namely F69 (S2), R166 (S1), and E167 (S1), hold significance for PLpro's deubiquitinating and deISGylating activities (Shin *et al.* 2020,  Klemm *et al*. 2020). Notably, the 166–167 region serves as an information-sharing site, linking residue 111. This is consistent across exact and approximate MI profiles (Fig. 8).

The 2D MI maps in PLpro uncover all residues that share information within the PLpro structure (Supplementary Fig. S3). The exact and multivariate MI maps closely align. The Ubl subdomain shows correlations within itself and with the thumb subdomain, which, in turn, correlates with itself and other subdomains. The fingers and palm subdomains are correlated with each other and with the thumb subdomain. The thumb subdomain acts as a bridge between the Ubl and the other two subdomains. The isotropic Gaussian MI map partially captures these regional correlations. The catalytic triad located at the convergence of thumb, palm, and fingers subdomains interacts with certain Ubl subdomain residues in the exact and multivariate Gaussian findings. The Ubl subdomain's role remains uncertain, yet potential allosteric interactions might exist between catalytic residues and specific Ubl residues (T4, D22, Q30, P46). R166 and E167 are pivotal, as mutating these residues leads to a loss of enzymatic activity. Notably, R166 shares information with distant residues like T4, D22, P46, H72, N128, K274, and I285, which also share correlations with the catalytic residues. F69 within the S2 binding region holds particular significance due to decreased enzymatic activity upon mutation. S2 may potentially allosterically interact with the Ubl, fingers, and palm residues. F69 exhibits pronounced correlations with distant residues—F8, H50, Y154, H175, A261, T277, C284, and S294- within its exact and multivariate Gaussian MI profiles. Notably, T277 and C284 are in proximity to the catalytic residues.

In contrast to the exact and multivariate Gaussian maps, GNM 1-all modes do not reveal information sharing between the Ubl and thumb domains. Instead, they highlight connections between the Ubl, fingers, and palm domains. However, GNM 2-all and other modes (3-all, 4-all, 5-all) exhibit correlations between the Ubl and thumb domains, while those between the Ubl, fingers, and palm are largely absent (Supplementary Fig. S4). This suggests that information sharing between the Ubl, fingers, and palm domains reflects long-term protease dynamics, whereas the dynamic connection between the Ubl and thumb signifies relatively faster motion. A 100 µs MD simulation of PLpro may possibly lack capturing slowest motion. Considering the PLpro's shape, the distant subdomains correlations naturally demand more time. The thumb subdomain links the Ubl domain to the fingers and palm domains. In the first 100 µs, the Ubl-thumb domains correlations emerge, evolving into distant Ubl-finger & palm domains correlations, causing the Ubl-thumb domains correlations fade. Not from the GNM's isotropic assumption, as the exact anisotropic map correlations appear in the GNM submodes. The exact isotropic and isotropic Gaussian maps align more with the exact anisotropic and GNM submodes, supporting the isotropic assumption's non-role in 100 µs exact map versus GNM 1-all modes differences.

Worth noting, the mutual information profiles for the Ubl domain in the isotropic Gaussian, GNM 2-all, GNM-3-all are more alike than the exact anisotropic map. Unlike other subdomains, the Ubl's mutual information evaluation is likely isotropic assumption-vulnerable, possibly due to its disruption of PLpro's spherical shape. Other subdomains form a stable sphere unaffected by the isotropic assumption, unlike the Ubl domain. The exact versus Gaussian isotropic differences might arise from the Gaussian approximation excluding orthogonal correlations.

### 3.8 Limitations of GNM

It is important to note that several instances exist where GNM may not be the optimal analysis method:

- Systems with substantial conformational changes: GNM relies on static structures and may not effectively capture large conformational changes, such as those observed in enzymes with significant substrate-induced movements.
- Systems with anisotropic dynamics: GNM assumes isotropic interactions between residues and may miss important correlations in proteins with pronounced directional fluctuations.
- Systems with complex allosteric mechanisms: GNM focuses on pairwise correlations and may not adequately capture the intricate higher-order interactions involved in complex allosteric pathways.
- Highly dynamic systems with fast functional timescales: GNM's coarse-grained nature might overlook rapid functional motions in highly dynamic systems.
- Systems with non-equilibrium dynamics: GNM assumes equilibrium dynamics and may not be suitable for systems undergoing non-equilibrium processes like active transport or protein-protein interactions.

## 4 Conclusion

In this study, we have explored the application of mutual information analysis for investigating allosteric connectivity, focusing on the accuracy and limitations of different approximations. In that, our goal is to determine optimal trajectory lengths that accurately capture long range dynamic correlations and evaluate various models, including exact anisotropic calculations, multivariate Gaussian and isotopic Gaussian approximations, isotropic Gaussian model, and GNM.

We have assessed the accuracy of different approximations across varies MD trajectory lengths. The multivariate Gaussian model demonstrates robust performance in representing mutual information profiles. In contrast, the isotropic Gaussian model, which neglects orthogonal correlations, shows limitations in capturing exact anisotropic mutual information profiles.

Our findings highlight the crucial role of an optimum trajectory length in capturing precise mutual information profiles that reveal long range dynamic information exchange patterns with respect to the maximum MD trajectory available, as illustrated with examples of Ubiquitin and PLpro. While noting that different time windows from several parallel MD trajectories possibly reveal various functionally plausible correlations, achieving long range correlations necessitates the sampling essential parts of the conformational/dynamic space to represent functional time scales. Therefore, the sufficiency of trajectory length is crucial for effective Gaussian approximation to accurately capture long-range information exchange patterns. This suggests the need for longer time scales, as the maximum trajectory lengths here may not represent the ground truth. Furthermore, the optimum trajectory length for approximating long-time dynamic behavior may differ for diverse proteins, depending on their intrinsic dynamics, which are influenced by topology and structural features (such as size and number of domains) that have been evolutionarily optimized for functional motions.

Finally, we have also explored the potential of GNM to represent mutual information profiles, offering a promising option due to its speed and applicability to crystal structures. Despite its isotropic nature, GNM proves effective in representing long-time behavior when the protein topology undergoes minimal conformational changes, as observed in Ubiquitin and PLpro. GNM also facilitates dissecting dynamics within mutual information frameworks, inferring relative timescales of information sharing patterns, and thus aiding in assessing MD simulation sufficiency.

Overall, we propose a compelling path for future investigations into dynamic allostery, aiming to unveil networks of function-specific characteristics and ultimately enhance our understanding and characterization of protein functional mechanisms.

## Supplementary Material

btae076_Supplementary_Data

## Data Availability

Mutual information codes and data are available at https://github.com/kemaldemirtas/prc-MI.git.
