# Supplementary material for: Dynamic correlations: exact and approximate methods for mutual information
Source: Bioinformatics. 2024 Feb 10;40(2):btae076. doi: 10.1093/bioinformatics/btae076 (PMC10898342; doi:10.1093/bioinformatics/btae076)
Supplement: btae076_Supplementary_Data [file btae076_supplementary_data.docx]

**DERIVATION OF GAUSSIAN BASED MUTUAL INFORMATION METHODS**

General relations for entropy, mutual information and interaction information:

The Shannon entropy expression, $H$ is

$$H=-k\left\langle\log p \right\rangle(1)$$

where *p* is the probability which in terms of fluctuations$\Delta\text{R}_{i}$of n residues it is $p\left(\Delta\text{R}_{1},\Delta\text{R}_{2},...,\Delta\text{R}_{n}\right)$. In Shannon’s notation, the log is to base 2 and the constant k is unity. When k is the Boltzmann constant and the log is the natural logarithm, $H$becomes identical with the thermodynamic entropy (See for example Callen et al. 1985). In the following, we will take k=1 and the natural logarithm.

Mutual information is defined as

$$I\left( {\Delta\boldsymbol{R}}_{i};{\Delta\boldsymbol{R}}_{j} \right)=\left\langle\log\left( \frac{p\left( {\Delta\boldsymbol{R}}_{i},{\Delta\boldsymbol{R}}_{j} \right)}{p\left( {\Delta\boldsymbol{R}}_{i} \right)p\left( {\Delta\boldsymbol{R}}_{j} \right)} \right) \right\rangle(2)$$

where, $\Delta\text{R}_{i}$is the instantaneous fluctuation of residue i, $p\left(\Delta\text{R}_{i},\Delta\text{R}_{j}\right)$ is the joint probability of $\Delta\text{R}_{i}$and $\Delta\text{R}_{j}$. Mutual information is the amount of information gained from the correlation of X and Y. Therefore it is a measure of the interaction of X and Y. It can be shown that $I\geq0$. If X and Y are independent, then $I=0$. For more detailed information on mutual information see Thomas and Cover (Cover et al. 1999).

In this study, we will consider the probabilities to be multivariate Gaussian. Accordingly,

$$p_{ij}\left( \Delta\boldsymbol{R} \right)=\left( 2\pi\right)^{-\frac{n}{2}}{(det\left\langle\Delta\boldsymbol{R\Delta}\boldsymbol{R}^{T} \right\rangle)}^{-\frac{1}{2}}\exp\left[ -\frac{1}{2}\Delta\boldsymbol{R}^{T}\left\langle\Delta\boldsymbol{R}{\Delta\boldsymbol{R}}^{T} \right\rangle^{-1}\Delta\boldsymbol{R} \right] (3)$$

where n is the dimension represented by the number of components of $\Delta\text{R}$ having n entries

$$\Delta\boldsymbol{R}=col\left[ {\Delta\boldsymbol{R}}_{1} {\Delta\boldsymbol{R}}_{2} \ldots{\Delta\boldsymbol{R}}_{n} \right] (4)$$

We now derive the average $\left\langle\ln p\left(\text{R}\right)\right\rangle$ for the multivariate Gaussian with n variables.

We write the exponent in Eq. 3 as

$$\Delta\boldsymbol{R}^{T}\left\langle\Delta\boldsymbol{R}{\Delta\boldsymbol{R}}^{T} \right\rangle^{-1}\Delta\boldsymbol{R}= \Delta\boldsymbol{R}^{T}\left\langle\Delta\boldsymbol{R}{\Delta\boldsymbol{R}}^{T} \right\rangle^{-1/2}\left\langle\Delta\boldsymbol{R}{\Delta\boldsymbol{R}}^{T} \right\rangle^{-1/2}\Delta\boldsymbol{R}(5)$$

And use the orthonormal transformation

$$\Delta\boldsymbol{r}= \left\langle\Delta\boldsymbol{R}\Delta\boldsymbol{R}^{T} \right\rangle^{-1/2}\Delta\boldsymbol{R}(6)$$

Rearranging the Gaussian expression and substituting into the Shannon equation leads to:

$$H=-\left\langle log\left\{ \left( 2\pi\right)^{-\frac{n}{2}}{(det\left\langle\Delta\boldsymbol{R\Delta}\boldsymbol{R}^{T} \right\rangle)}^{-\frac{1}{2}}\exp\left[ -\frac{1}{2}{\Delta\boldsymbol{R}}^{T}\left\langle\Delta\boldsymbol{R\Delta}\boldsymbol{R}^{T} \right\rangle^{-1}\Delta\boldsymbol{R} \right] \right\} \right\rangle=\frac{n}{2}\log\left( 2\pi\right)+\frac{1}{2}\log\left( det\left\langle\Delta\boldsymbol{R\Delta}\boldsymbol{R}^{T} \right\rangle\right)+\frac{1}{2}\left\langle{\Delta\boldsymbol{R}}^{T}\left\langle\Delta\boldsymbol{R\Delta}\boldsymbol{R}^{T} \right\rangle^{-1}\Delta\boldsymbol{R} \right\rangle=\frac{n}{2}\log\left( 2\pi\right)+\frac{1}{2}\log\left( det\left\langle\Delta\boldsymbol{R\Delta}\boldsymbol{R}^{T} \right\rangle\right)+\frac{1}{2}\left\langle{\Delta\boldsymbol{r}}^{T}\Delta\boldsymbol{r} \right\rangle=\frac{1}{2}\log\left( det\left\langle\Delta\boldsymbol{R\Delta}\boldsymbol{R}^{T} \right\rangle\right)+\frac{n}{2}\log\left( 2\mu\right)+\frac{n}{2} =\frac{1}{2}\log\left( det\left\langle\Delta\boldsymbol{R\Delta}\boldsymbol{R}^{T} \right\rangle\right)+\frac{n}{2}\log\left( 2\mu e \right) (7)$$

Or,

$$H\left( \Delta\boldsymbol{R} \right)=\frac{1}{2}\log\left( {det\left\langle\Delta\boldsymbol{R\Delta}\boldsymbol{R}^{T} \right\rangle}_{n} \right)+ \frac{n}{2}\log\left( 2\pi e \right) (8)$$

In Eqs 7 and 8, n represents the dimension of the Gaussian. If the fluctuation of each residue is represented by its three Cartesian components, $\Delta X$, $\Delta Y$, $\Delta Z$ then n in Eqs 3 and 4 is 2, 7 and 8 for one, two and three residues, respectively. In expanded form, for one residue n=3

$$\Delta\boldsymbol{R}=col\left[ {\Delta\boldsymbol{R}}_{i} \right]=col\left[ {\Delta X}_{i} {\Delta Y}_{i} {\Delta Z}_{i} \right] (9)$$

For two residues, i, j, n=6 and

$$\Delta\boldsymbol{R}=col\left[ {\Delta\boldsymbol{R}}_{i} {\Delta\boldsymbol{R}}_{j} \right]=col\left[ {\Delta X}_{i} {\Delta Y}_{i} {\Delta Z}_{i} {\Delta X}_{j} {\Delta Y}_{j} {\Delta Z}_{j} \right] (10)$$

Using Eq. 3, mutual information in terms of entropy is written as

$$I\left( {\Delta\boldsymbol{R}}_{i};{\Delta\boldsymbol{R}}_{j} \right)=H\left( {\Delta\boldsymbol{R}}_{i} \right)+H\left( {\Delta\boldsymbol{R}}_{j} \right)-H\left( {\Delta\boldsymbol{R}}_{i},{\Delta\boldsymbol{R}}_{j} \right) (11)$$

Substituting from Eq. 8 leads to the general expression for mutual information as

$$I\left( {\Delta\boldsymbol{R}}_{i};{\Delta\boldsymbol{R}}_{j} \right)=-\frac{1}{2}\log\left( 1-\frac{det\left\langle\Delta\boldsymbol{R\Delta}\boldsymbol{R}^{T} \right\rangle}{det\left\langle{\Delta\boldsymbol{R}}_{i}{\Delta\boldsymbol{R}}_{i}^{T} \right\rangle det\left\langle{\Delta\boldsymbol{R}}_{j}{\Delta\boldsymbol{R}}_{j}^{T} \right\rangle} \right) (12)$$

or

$$I\left( {\Delta\boldsymbol{R}}_{i};{\Delta\boldsymbol{R}}_{j} \right)=-\frac{1}{2}\log\left( 1-\frac{\left\langle{{\Delta\boldsymbol{R}}_{i}\Delta\boldsymbol{R}}_{j}^{T} \right\rangle^{2}}{\left\langle\left( {\Delta\boldsymbol{R}}_{i} \right)^{2} \right\rangle\left\langle\left( {\Delta\boldsymbol{R}}_{j} \right)^{2} \right\rangle} \right) (13)$$

where $\Delta\text{R}$is given by Eq. 10. This is the general multivariate Gaussian expression for mutual information.

When the fluctuations are isotropic, the matrix $\left\langle\Delta\text{R}\Delta\text{R}^{T}\right\rangle_{n}$may be replaced by $\left\langle\Delta\text{R}^{T}\Delta\text{R}\right\rangle_{n}$which eliminates all the off-diagonal elements such as $\left\langle\Delta X_{i}\Delta Y_{j}\right\rangle$etc. Equation 13 then take the form

$$I\left( {\Delta\boldsymbol{R}}_{i};{\Delta\boldsymbol{R}}_{j} \right)=-\frac{1}{2}\log\left( 1-\frac{\left\langle{\Delta\boldsymbol{R}}_{i}^{T}.{\Delta\boldsymbol{R}}_{j} \right\rangle^{2}}{\left\langle\left( {\Delta\boldsymbol{R}}_{i} \right)^{2} \right\rangle^{\frac{1}{2}}\left\langle\left( {\Delta\boldsymbol{R}}_{j} \right)^{2} \right\rangle^{\frac{1}{2}}} \right) (14)$$

Equation 14 now misses the off-diagonal elements in $\left\langle\Delta\text{R}\Delta\text{R}^{T}\right\rangle_{n}$and the GNM obtains this average from the inverse of the contact matrix.

**REFERENCES**

Callen H.B. et al. (1985) Thermodynamics and an introduction to thermostatistics. Second ed., *Wiley*.

Cover, T. M. et al. (1999) *Elements of information theory*. John Wiley & Sons.

**TABLES**

**Table S1:** Number of replicas of Ubiquitin used for each trajectory length

| **Length** | **Number of replicas** |
| --- | --- |
| 1.4 ns | 101 |
| 1.6 ns | 101 |
| 1.8 ns | 101 |
| 2 ns | 101 |
| 3 ns | 101 |
| 4 ns | 101 |
| 5 ns | 101 |
| 10 ns | 51 |
| 50 ns | 51 |
| 100 ns | 21 |
| 500 ns | 21 |
| 1 µs | 11 |
| 5 µs | 6 |
| 10 µs | 6 |
| 100 µs | 2 |

**Table S2:** Number of replicas of PLpro used for each trajectory length

| **Length** | **Number of replicas** |
| --- | --- |
| 50 ns | 20 |
| 100 ns | 20 |
| 150 ns | 20 |
| 200 ns | 20 |
| 250 ns | 20 |
| 300 ns | 20 |
| 350 ns | 20 |
| 400 ns | 20 |
| 500 ns | 20 |
| 1 µs | 10 |
| 5 µs | 10 |
| 10 µs | 5 |
| 25 µs | 4 |

**FIGURES**


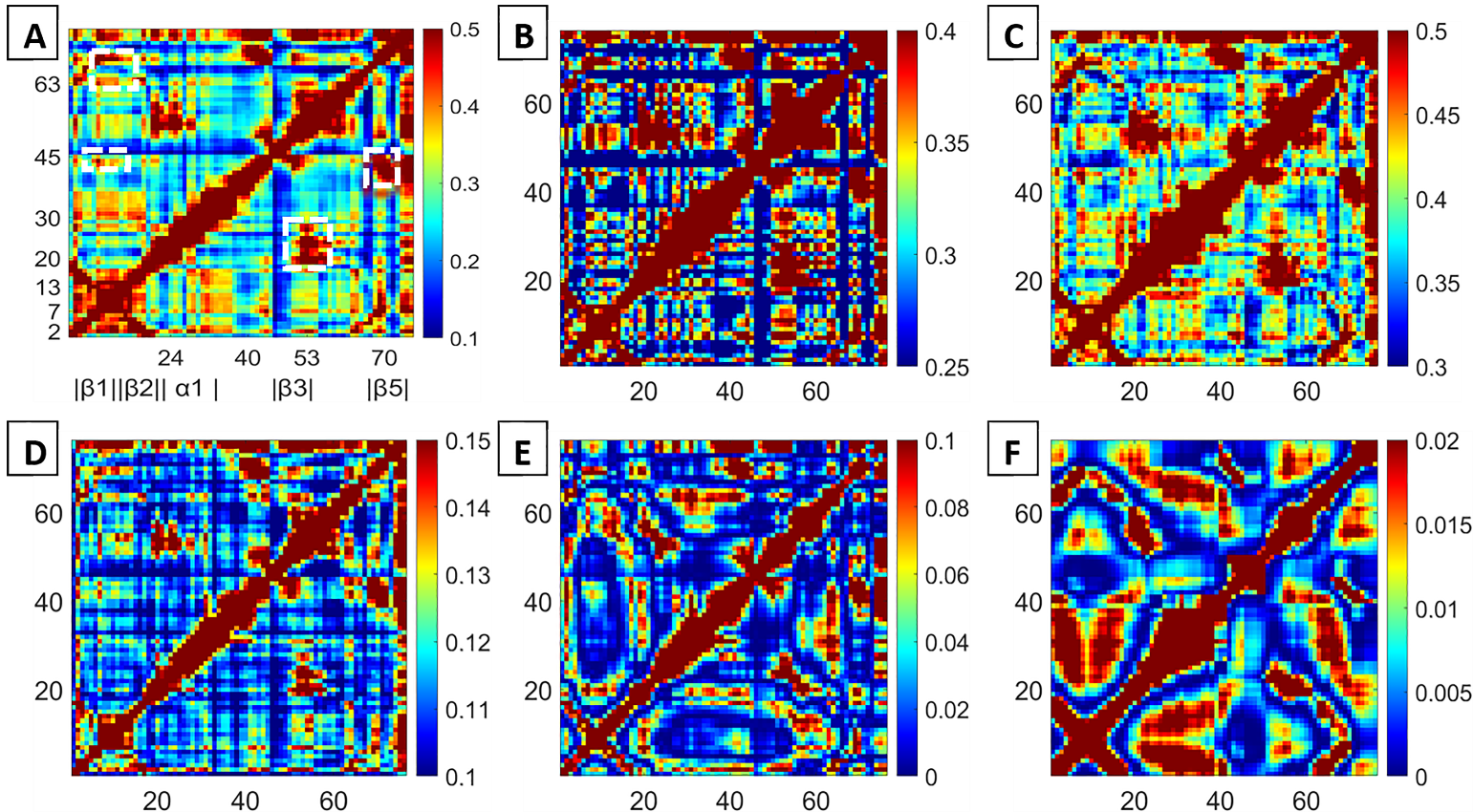


**Figure S1:** 2D MI maps of Ubiquitin. The exact anisotropic MI of 1 ms (A), the exact anisotropic MI of 5 µs trajectory (B), the multivariate Gaussian MI evaluation of 5 ns trajectory (C), the exact isotropic MI of 1 µs trajectory, (E) the isotropic Gaussian MI of 500 ns (E), GNM 1-all MI (F).

**
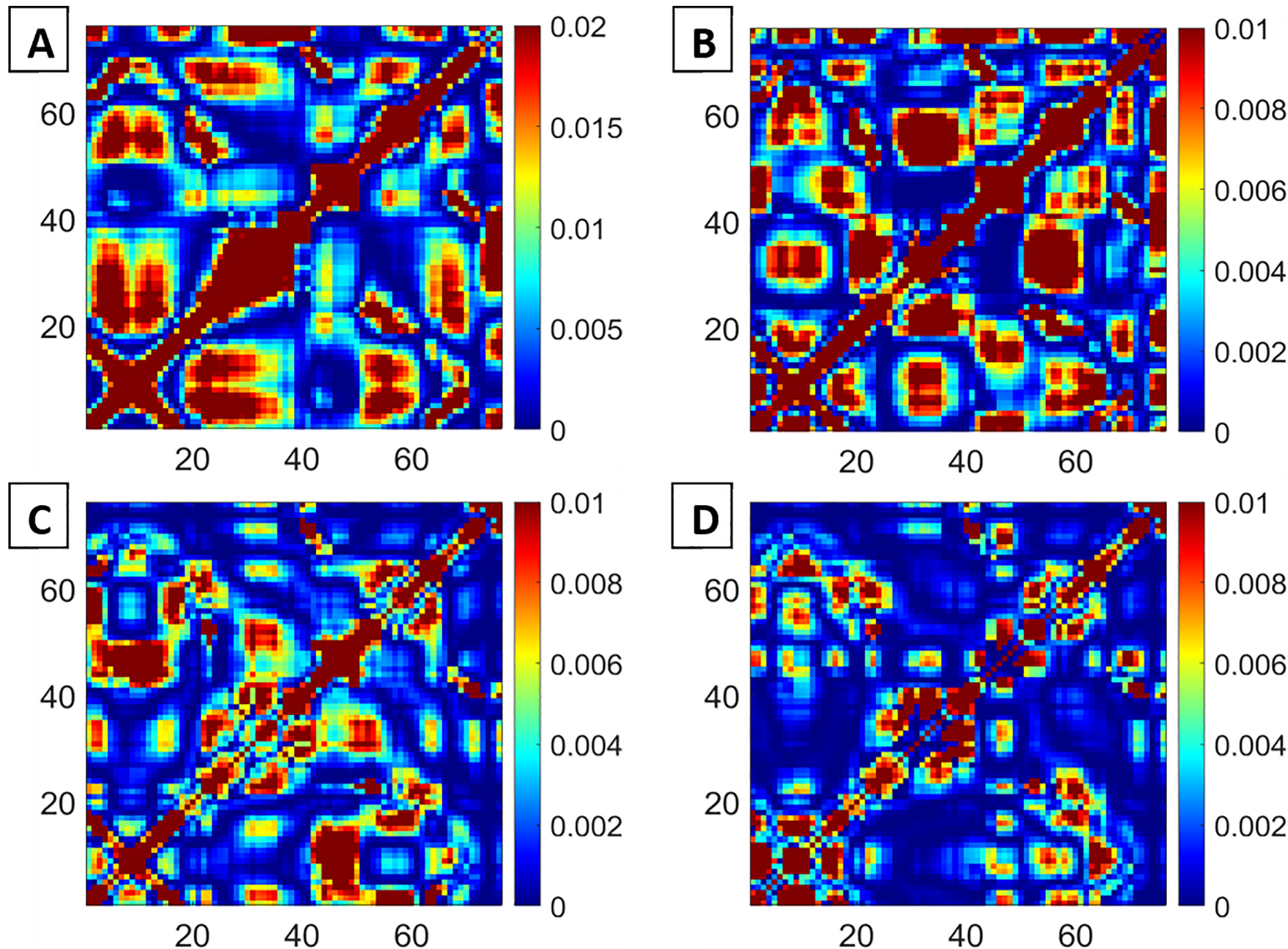
**

**Figure S2:** 2D GNM MI in the subsets of slow modes of Ubiquitin. GNM 2-all (A), GNM 3-all (B), GNM 4-all (C), (D) GNM 5-all (D).

**
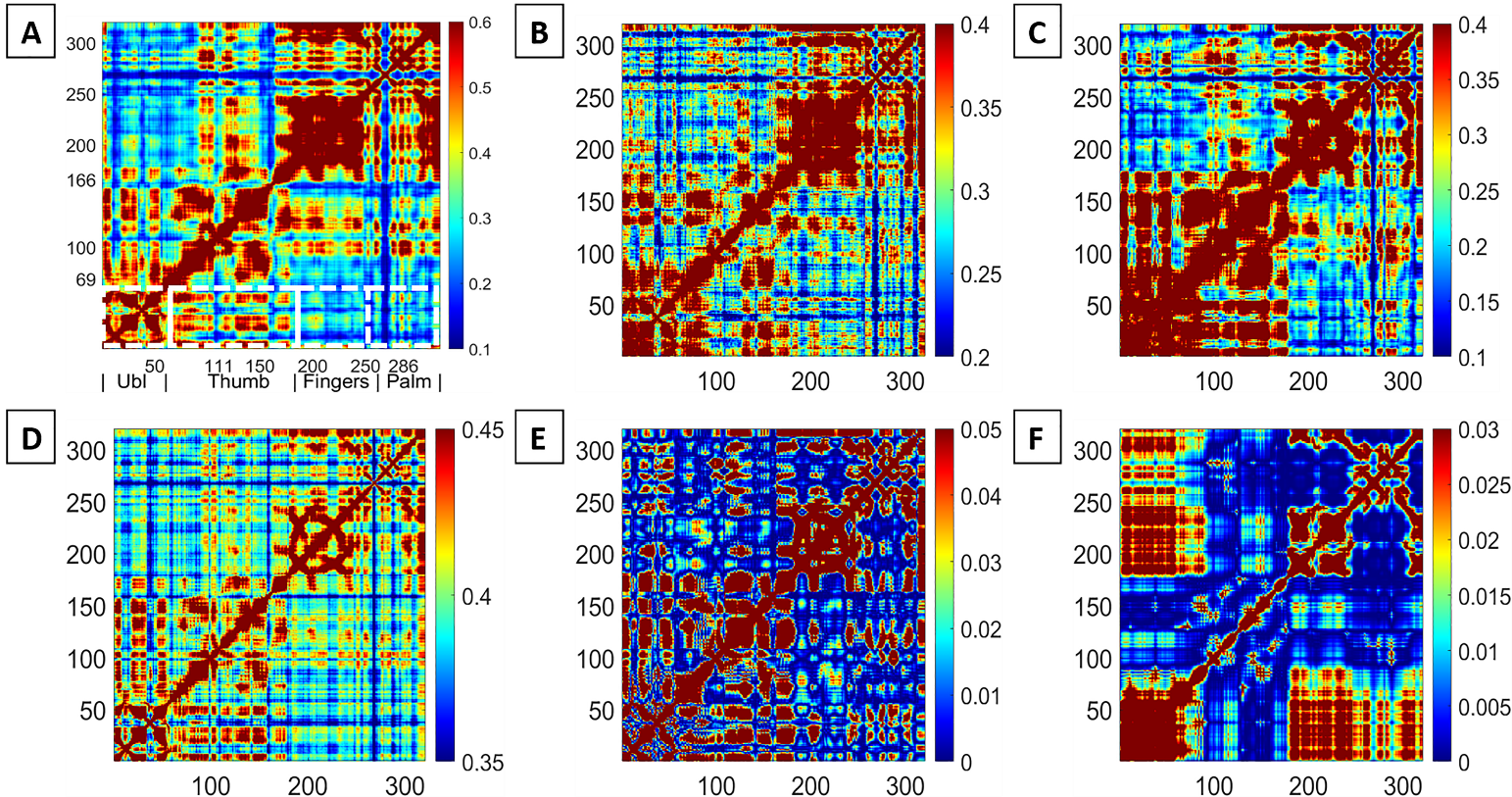
Figure S3:** 2D MI maps of PLpro. The anisotropic MI of 100 µs (A), the exact anisotropic MI of 1 µs (B), the multivariate Gaussian MI of 350 ns (C), the exact isotropic MI of 1 µs, the isotropic Gaussian MI of 300 ns (D), GNM 1-all MI.

**
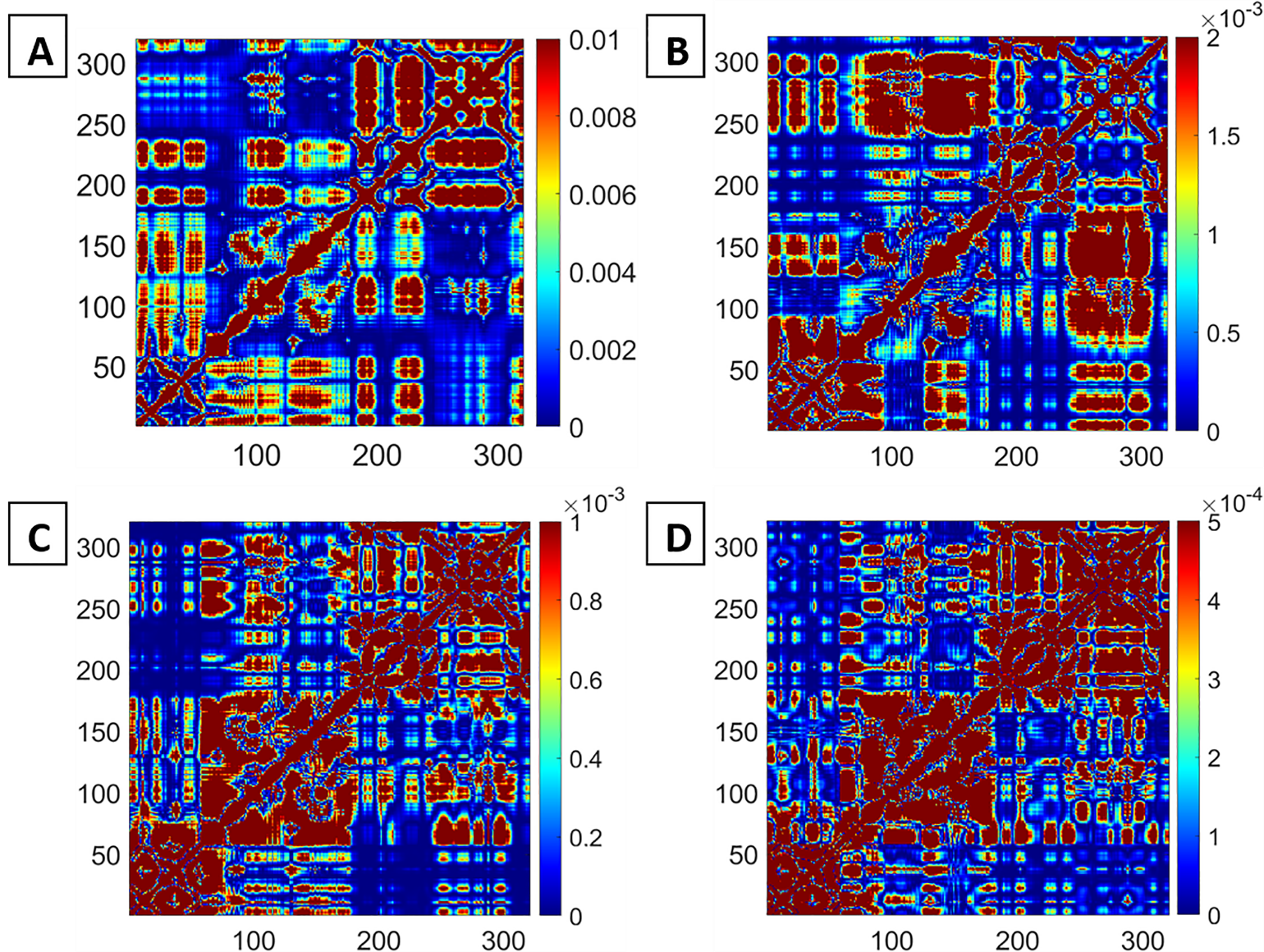
**

**Figure S4:** 2D GNM submodes MI maps of 1UBQ. (A) GNM 2-all, (B) GNM 3-all, (C) GNM 4-all, (D) GNM 5-all.

**
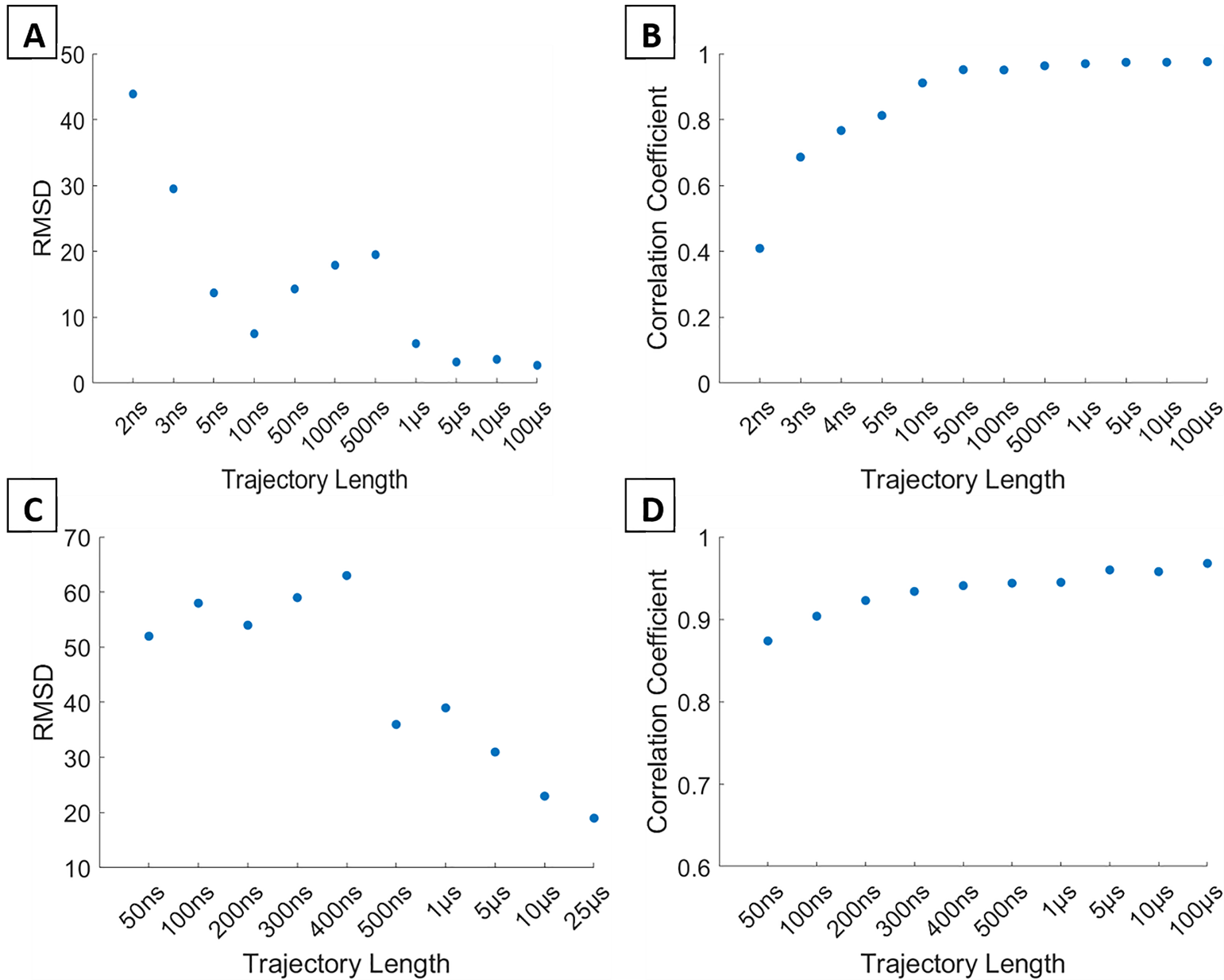
Figure S5:** Exact Anisotropic MI. RMSD and correlation coefficient values between the exact anisotropic MI profile at various trajectory lengths and the maximum trajectory length are respectively shown for all residue pairs of Ubiquitin (A, B) and PLpro (C, D). The maximum trajectory length is 1ms for Ubiquitin and 100 µs for PLpro.


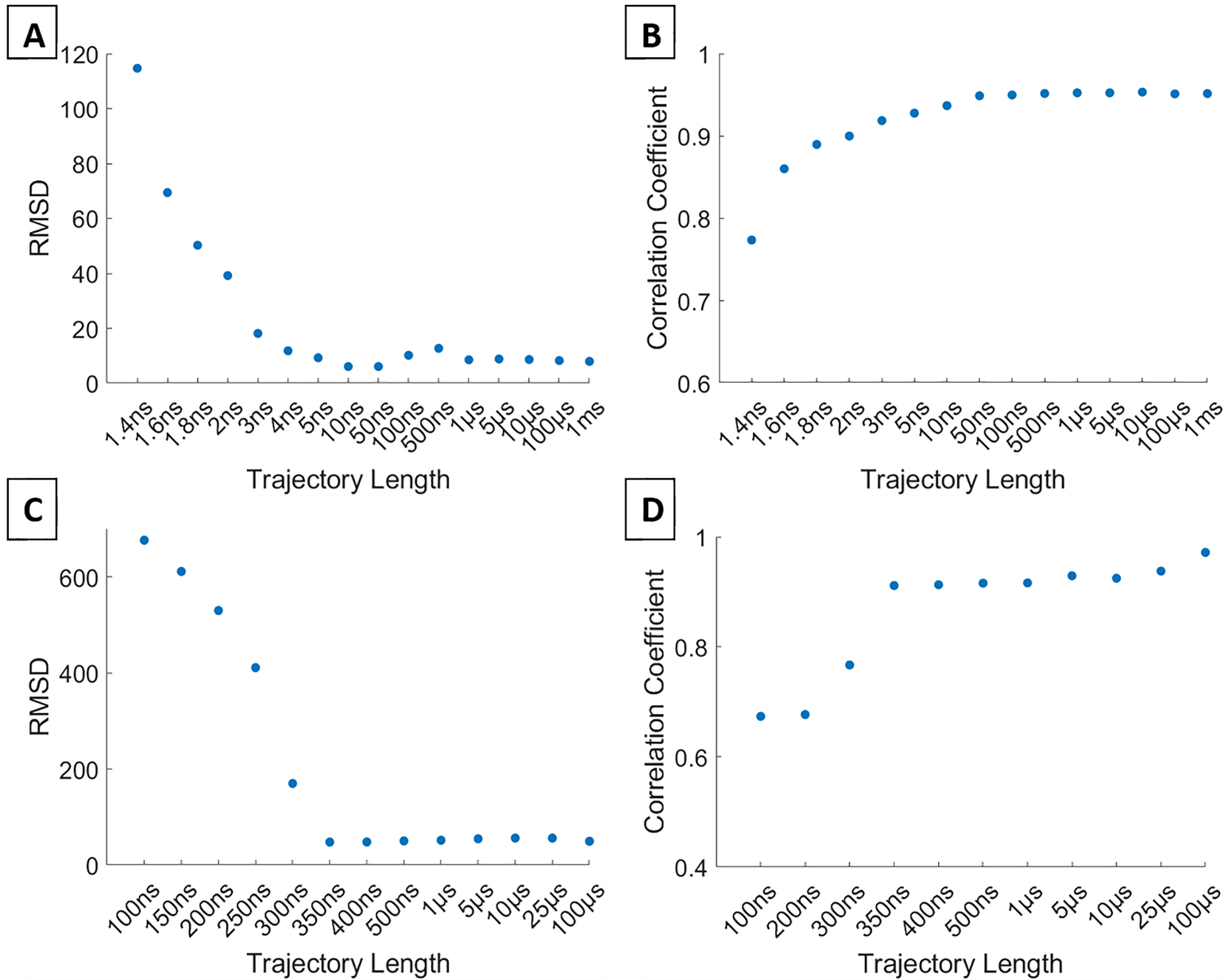


**Figure S6:** Multivariate Gaussian MI. RMSD and correlation coefficient values between the multivariate Gaussian MI profiles at various trajectory lengths and the exact anisotropic MI profile at the maximum trajectory length are respectively shown for all residue pairs of Ubiquitin (A, B) and PLpro (C, D).

**
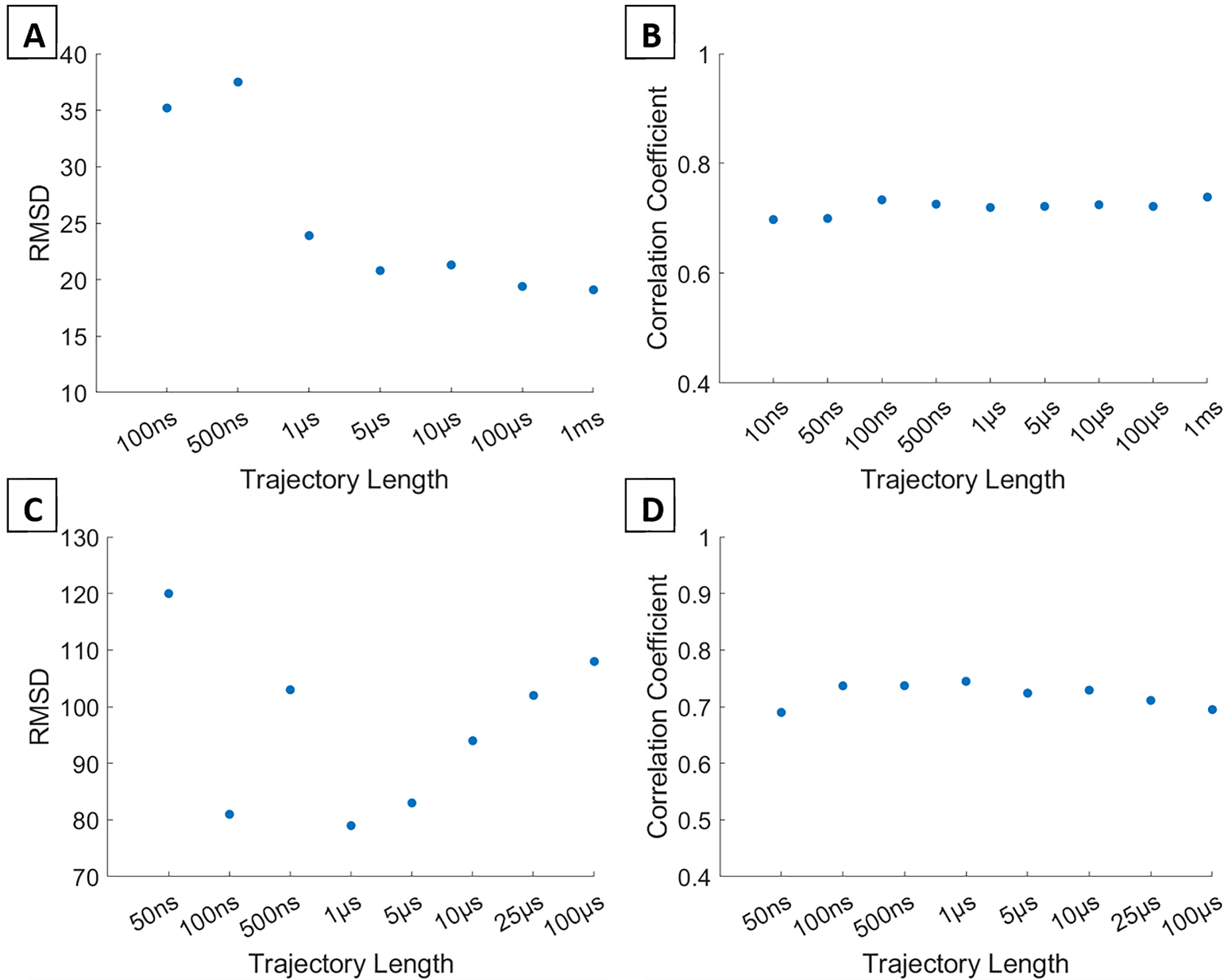
Figure S7:** GNM MI. RMSD and correlation coefficient values between the GNM MI profiles and the exact anisotropic MI profile at various trajectory lengths are respectively shown for all residue pairs of Ubiquitin (A, B) and for PLpro (C, D).
